# Multimodality MRI-based radiomics for aggressiveness prediction in papillary thyroid cancer

**DOI:** 10.1186/s12880-022-00779-5

**Published:** 2022-03-24

**Authors:** Zedong Dai, Ran Wei, Hao Wang, Wenjuan Hu, Xilin Sun, Jie Zhu, Hong Li, Yaqiong Ge, Bin Song

**Affiliations:** 1 Department of Radiology, Minhang Hospital, Fudan University, 170 Xinsong Road, Shanghai, 201199 People’s Republic of China; 2 GE Healthcare, Shanghai, People’s Republic of China

**Keywords:** Radiomics, Papillary thyroid carcinoma, Aggressiveness, Multimodality MRI, Sparse representation

## Abstract

**Objective:**

To investigate the ability of a multimodality MRI-based radiomics model in predicting the aggressiveness of papillary thyroid carcinoma (PTC).

**Methods:**

This study included consecutive patients who underwent neck magnetic resonance (MR) scans and subsequent thyroidectomy during the study period. The pathological diagnosis of thyroidectomy specimens was the gold standard to determine the aggressiveness. Thyroid nodules were manually segmented on three modal MR images, and then radiomics features were extracted. A machine learning model was established to evaluate the prediction of PTC aggressiveness.

**Results:**

The study cohort included 107 patients with PTC confirmed by pathology (cross-validation cohort: n = 71; test cohort: n = 36). A total of 1584 features were extracted from contrast-enhanced T1-weighted (CE-T1 WI), T2-weighted (T2 WI) and diffusion weighted (DWI) images of each patient. Sparse representation method is used for radiation feature selection and classification model establishment. The accuracy of the independent test set that using only one modality, like CE-T1WI, T2WI or DWI was not particularly satisfactory. In contrast, the result of these three modalities combined achieved 0.917.

**Conclusion:**

Our study shows that multimodality MR image based on radiomics model can accurately distinguish aggressiveness in PTC from non-aggressiveness PTC before operation. This method may be helpful to inform the treatment strategy and prognosis of patients with aggressiveness PTC.

## Introduction

Thyroid cancer is one of the most common malignant tumor in the head and neck [1]. The histological types of this disease include papillary carcinoma, myeloid carcinoma and follicular carcinoma. This disease is not easy to be found at the time of onset, and its slow course causes most patients to be accompanied by aggressiveness. There are 15 subtypes of papillary carcinoma [2], and the histological characteristics, imaging characteristics and prognosis of different subtypes are different [3]. Accompanied by local invasion, extraglandular invasion, lymph node metastasis and distant organ metastasis, when any of the above conditions occurs, it is determined as invasive thyroid cancer [4, 5]. The invasive subtypes considered in the 2015 American Thyroid Association Management Guidelines for Adult Patients [6] include high cell subtype, columnar cell subtype and shoe nail subtype. Some studies [7] show that the diffuse sclerosis subtype also belongs to aggressiveness subtype. At present, surgical resection is needed for most aggressive thyroid cancer, and the prognosis is relatively poor. Therefore, early diagnosis and identifying whether thyroid cancer is aggressive is of great significance. Nowadays, there are many research mechanisms for aggressive thyroid cancer, among which pathological tissue biopsy is the recognized gold standard for diagnosis [8]. However, whether the puncture results are satisfactory is affected by many factors, such as the size and location of thyroid nodules, the presence or absence of calcification and liquefaction in the nodes, also the experience of operators and cytologists. And in the implementation process, it will also cause a certain degree of trauma to patients and increase the risk of bleeding and infection. Therefore, it is of great significance to develop a non-invasive method to automatically identify the aggressiveness of thyroid cancer.

At present, the conventional imaging examination methods of thyroid cancer include ultrasound, computed tomography (CT) and magnetic resonance imaging (MRI). Ultrasound is the preferred imaging examination method in the diagnosis of thyroid cancer, which has the characteristics of fast, real-time dynamic, no radiation and high resolution. However, due to the influence of neck bone and air, it is kind of difficult to distinguish the difference between blood flow and the echo of surrounding tissue. And the accuracy of ultrasound in evaluating deep neck structure still needs more researches [9, 10]. So when it comes to an effective remedy for weaknesses in ultrasound, people naturally think of CT and MRI. In terms of CT, it can show the relationship between the anatomical location, morphology, and surrounding tissues of thyroid cancer, however, it is not without faults, like radiation, which limits the application scope of clinicians. While MRI has excellent sensitivity in the diagnosis of thyroid cancer because of its high resolution to soft tissue. Through multi sequence scanning of the nidi, clear images of the nidi and adjacent tissues can be obtained, and the influence of subcutaneous fat on the image quality of patients can be avoided.

Radiomics comes from computer-aided detection or diagnosis (CAD), which combine image quantitative analysis with machine learning method [11]. At present, the basic function of radiomics is to analyze the tumor region of interest (ROI) quantitatively through a large number of imaging features, so as to provide valuable diagnosis, prognosis or prediction information. And the purpose of radiomics is to explore and use these information resources to develop suitable radiomics models for diagnosis, prediction, or prognosis, to support personalized clinical decision-making and improve individualized treatment options. MRI excels in soft tissue imaging and can provide high contrast structural and functional information. Diffusion weighted imaging (DWI) and dynamic contrast-enhanced magnetic resonance imaging (DCE-MRI) can reflect tissue cell structure and angiogenesis. Through the acquisition of these images, more effective imaging features can be extracted.

However, there are few reports on the application of radiomics to evaluate aggressiveness in papillary thyroid carcinoma (PTC) based on MRI, which indicates that there is a certain space for research in this field of knowledge. Therefore, MRI based radiomics technique may provide a noninvasive and accurate method for predicting thyroid aggressiveness in patients with PTC. This work aims to evaluate whether it is possible to detect thyroid aggressiveness in PTC by using multimodality MRI based radiomics method.

## Method

### Patients

The current retrospective trial evaluated patients with continuous thyroid nodules first detected by ultrasound from January 2018 to March 2019. According to the American Society of Radiology thyroid imaging, reporting and data system [12], the grade of tumor was TR3-TR5. All patients underwent multi parameter MRI, followed by thyroid surgery, subtotal or total thyroidectomy within 1 week after MRI. PTC was confirmed by pathology. The exclusion criteria were: (1) pathological diagnosis did not reflect PTC; (2) Tumor size < 5 mm; (3) There was no correlation between the pathological data of tumor specimens and the results of magnetic resonance imaging; (4) Poor image quality. Finally, 107 cases were evaluated.

The study was approved by our local institutional ethics committee.

### MRI acquisition

All patients were scanned on the excite HD 1.5 T scanner (GE Healthcare, USA), which included an 8-channel special neck surface coil using the same scanning protocol. The applied parameters were as follows: axial T2-weighted (T2WI) fast recovery fast spin-echo with fat suppression with echo time (TE) of 85 ms, repetition time (TR) of 1280 ms, and slice thickness of 4–5 mm, matrix of 288 × 192, spacing of 1 mm, field of view (FOV) of 18 cm, and a number of excitations (NEX) of 4; contrast-enhanced axial T1WI (CE-T1WI) with multiphase utilizing a fast-spoiled gradient recalled echo sequence, which TE of 1.7 ms, TR of 5.7 ms, matrix of 192 × 256, FOV of 14 cm, and NEX of 1;DWI with a single-shot echo planar imaging (EPI) sequence, with minimal TE, and TR of 6550 ms, slice thickness of 4–5 mm, matrix of 128 × 128, spacing of 0.5 mm, FOV of 14 cm, and NEX of 4 (b value, 800 s/mm^2^). The magnevist contrast agent from Bayer healthcare was administered intravenously at 3 ml / S (0.2 ml/kg), and then rinsed with 20 ml normal saline. Scanning was performed at 30, 60, 120, 180, 240 and 300 s after contrast agent administration to obtain images of six stages including breath-holds. The spatial saturation band was used to remove signals generated by covering fat and surrounding tissues.

### Histopathologic analysis

Surgical tumor cases were evaluated and analyzed by experienced pathologists who have been engaged in relevant research for more than 10 years. Tumor specimens were paraffin embedded and sectioned, and stained with hematoxylin eosin (H&E). And then the established criteria were used to assess thyroid aggressive characteristics [13, 14]. Finally, all patients were divided into non-aggressiveness group and aggressiveness group.

### MRI radiomics

#### Tumor segmentation

**Fig. 1:**
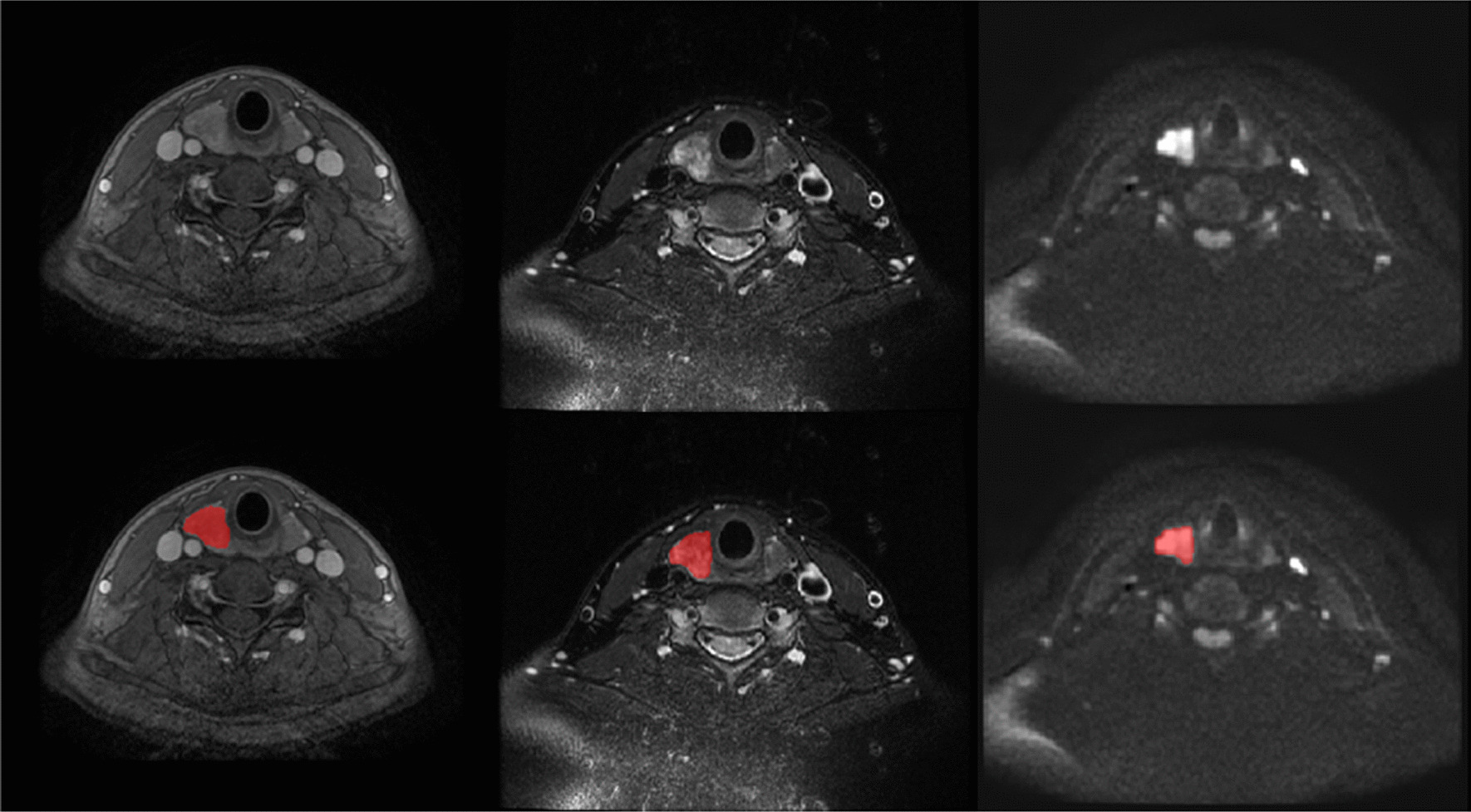
Segmentation results of CE-T1WI, T2WI and DWI modalities, the red area in the second line represents the segmentation result

Tumor segmentation is the key step of subsequent high-throughput feature extraction and quantitative analysis. In this paper, the segment editor part of 3D slicer software is used to segment the focus area of thyroid cancer. It is a module for segmentation, which can subdivide and depict the region of interest. Two senior radiologists manually marked them separately and discussed repeatedly to obtain the final results in case of disagreement. The largest tumor of each patient was selected in order to reduce the potential differences of multiple tumors in the same individual, which greatly improve the applicability of the results. And to reduce the impact of segmentation accuracy on model performance, we give up including the cases with disagreement segmentation in the experimental dataset. Figure 1 shows the results of three modal MRI, the first row is the images of three MRI modalities, and the second row corresponds to the segmentation results.

#### Radiomics feature extraction

Radiomics feature is the basic attribute description of class, and feature extraction is the basis of classification work. In this paper, we extract a total of 1584 features from CE-T1WI, T2WI and DWI modalities, with 528 for each modality. The 528 features include: 18 intensity features, 15 shape features, 39 texture features (8 GLCM, 13 GLRLM, 13 GLSZM and 5 NGTDM) and 456 (8 wavelet submaps * 57 intensity and texture features) wavelet features. More details about these features please refer to Appendix 1. And this part of work was completed by MATLAB.

#### Radiomic feature selection

The problem considered in feature selection is to make the features sparse, that is, some redundant features are removed through this step, so as to reduce the computational cost. The input factors of the model are reduced, and the input–output relationship established by the model will be clearer, so the interpretability of the model can be improved. In this work, sparse representation is used to select a few crucial features for the following classification. The sparse representation-based feature selection model can be written as:

1 $${\hat{\text{w}}} = \mathop {{\text{argmin}}}\limits_{{{\rm w}}} {\text{y}} - {\text{Fw}}\left\| {_{2}^{2} + {\upgamma }} \right\|{\text{w}}_{0}$$

where $\mathrm{y}\in {\mathrm{R}}^{\mathrm{m}}$ is the training sample label, $\mathrm{m}$ is the number of training samples, $\mathrm{F}=[{\mathrm{f}}_{1},{\mathrm{f}}_{2}\cdots {\mathrm{f}}_{\mathrm{m}}{]}^{\mathrm{T}}\in {\mathrm{R}}^{\mathrm{m}\times 2\mathrm{K}}$ is the training sample feature set, $\upgamma$ is the sparse control parameter. The absolute value of each element in the representation coefficient $\mathrm{w}$ indicates the importance of the corresponding feature. Once the $\mathrm{w}$ has been calculated, we sort the features in descending order of importance according to the corresponding absolute value of the elements in $\mathrm{w}$. Finally, we select the optimal subset of features using a sequential advance method based on cross-validation (on the cross-validation set). Specifically, the first 5 features are selected as the initial feature subset, and then the 6th to 100th features are put into the subset in turn. And the accuracy of cross-validation is calculated whenever the feature subset is updated. The subset with the highest accuracy is selected as the optimal subset.

#### Model construction and validation

Model building is the herald of the data analysis stage. According to the results of feature selection in the previous step, we use a sparse representation method to establish a prediction model for the classification of aggressiveness and non-aggressiveness thyroid cancer. Specifically, suppose $\mathbf{F}= [{\mathbf{F}}_{1}\cdots {\mathbf{F}}_{\mathbf{c}}{\cdots \mathbf{F}}_{\mathbf{C}}]$ denotes the feature set of training samples from $\mathbf{C}$ classes, and ${\mathbf{F}}_{\mathbf{c}}$ is the sample feature set of class c. The first step of sparse representation can be formulated as:

2 $$\left\{{\varvec{\Psi}},{\varvec{\Phi}}\right\}=\underset{{\varvec{\Phi}},{\varvec{\Psi}}}{\mathrm{argmin}}{\sum }_{\mathrm{c}-1}^{\mathrm{C}}{\Vert {\mathbf{F}}_{\mathbf{c}} -{{\varvec{\Psi}}}_{\mathbf{c}}{{\varvec{\Phi}}}_{\mathbf{c}}{\mathbf{F}}_{\mathbf{c}} \Vert }_{\mathrm{F}}^{2}+\uplambda {\Vert {{\varvec{\Phi}}}_{\mathbf{c}}\overline{{\mathbf{F} }_{\mathbf{c}}}\Vert }_{\mathrm{F}}^{2},\mathrm{ s}.\mathrm{t}.{\Vert {\mathrm{\varphi }}_{\mathrm{q}}\Vert }_{2}^{2}\le 1$$

where is $\uplambda$ a scalar constant, $\overline{{\mathbf{F} }_{\mathbf{c}}}$ is the complementary matrix of ${\mathbf{F}}_{\mathbf{c}}$. Dictionary pair ${\varvec{\Psi}}$ and ${\varvec{\Phi}}$ are used to reconstruct and code $\mathbf{F}$, respectively. ${\mathrm{\varphi }}_{\mathrm{q}}$ is an atom of dictionary ${\varvec{\Psi}}$. When the dictionary pair ${\varvec{\Psi}}$ and ${\varvec{\Phi}}$ are learned, the classification model can be formulated as:

3 $${\mathrm{l}}_{\mathrm{i}}=\underset{\mathrm{c}}{\mathrm{argmin}}{\Vert {\mathrm{f}}_{\mathrm{i}}-{{\varvec{\Psi}}}_{\mathrm{c}}{{\varvec{\Phi}}}_{\mathrm{c}}{\mathrm{f}}_{\mathrm{i}}\Vert }_{2},\mathrm{ c}\in [1,\cdots ,\mathrm{ C}]$$

where ${\mathrm{l}}_{\mathrm{i}}$ is the class label of testing case $\mathrm{i}$, and ${\mathrm{f}}_{\mathrm{i}}$ is the feature of $\mathrm{i}$.

In our experiments, $\upgamma$ and $\uplambda$ were set to 0.1 and 0.01, respectively. The 107 cases were randomly divided into cross-validation (71) and testing (36) sets in a ratio of about 2: 1. The cross-validation set was used for feature selection in advance. When the number of features is determined, we directly use the cross-validation set to establish a sparse representation classification model and test the testing set on it. We compare the classification performance of each modality as well as the combination of three modalities. Here we use the simplest modality combination method, that is, direct concatenating the features of three modalities. The classification models were evaluated by calculating the subject operating characteristic curve (ROC), accuracy (ACC), sensitivity (SEN), specificity (SPE), negative predictive value (NPV) and positive predictive value (PPV).

## Results

### Patient feature and selection of the study cohort

A total of 107 patients were evaluated. According to the pathological results, they were classified into aggressiveness group and non-aggressiveness group. Among them, 51 patients were aggressiveness group, with an average age of 42.37 ± 14.27 years (12–73 years), and 56 patients were non- aggressiveness group, with an average age of 46.68 ± 13.86 years (22–77 years). Table 1 summarizes the clinical characteristics of PTC cases registered in this study. In addition to age, gender, the lesion diameter, location, metastasis and multifocal cancer were also included in our study. The gender, lesion diameter and LN metastasis were statistically significant, which was consistent with the results of previous studies. The cross-validation cohort including 71 cases, while the test cohort including 36 cases.

**Table 1:** Patient features in the aggressiveness and non-aggressiveness groups

	Aggressiveness (n = 51)	Non-aggressiveness (n = 56)	*P*-value
Age(years)	42.37 ± 14.27	46.68 ± 13.86	0.979
*Sex*			
Female	35	45	0.006
Male	16	11	
Diameter(mm)	13.08 ± 6.44	9.36 ± 3.86	0.001
*Location*			
1	28	32	0.443
2	1	2	
3	22	22	
*LN metastasis*			
Yes	31	0	0
No	20	56	
Multi-lesions			
Yes	12	11	0.938
No	39	45	

The *p*-values was calculated by independent sample t-test

### Feature selection

**Fig. 2:**
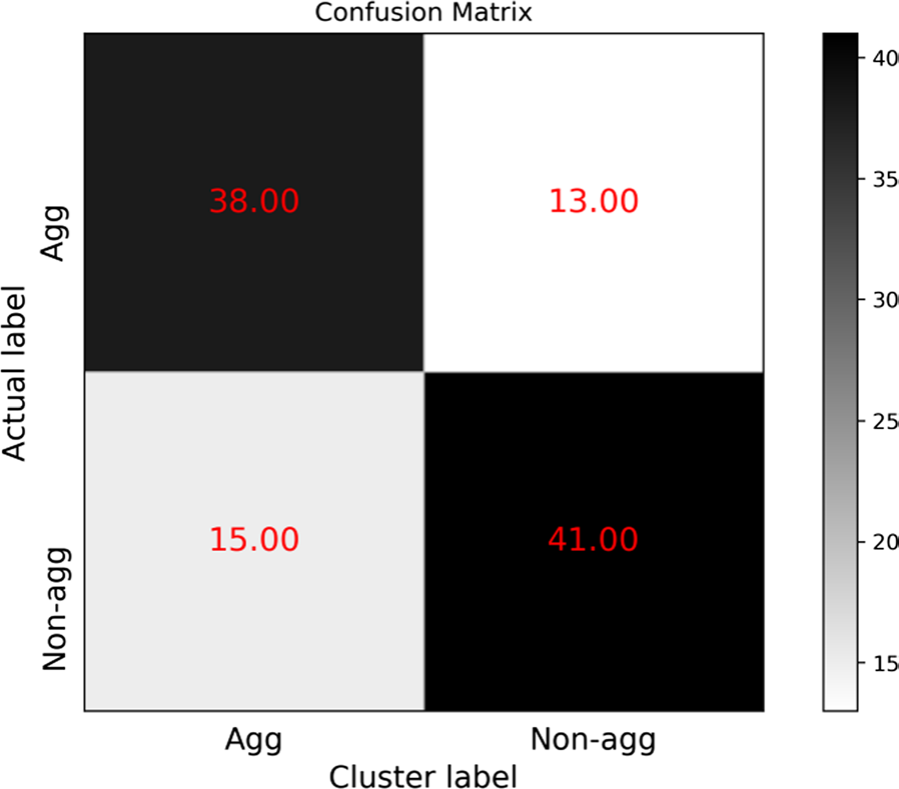
Confusion matrix of the clustering results. (Agg is the abbreviation of aggressiveness)

A total of 528 high-throughput features were extracted from CE-T1WI, T2WI and DWI modalities, respectively. In order to verify the effectiveness of these features, we first selected the features with *P* < 0.001 (with extremely significant statistical significance) by comparing the *P* values of t-test, and then performed unsupervised clustering on these features. Figure 2 shows the confusion matrix of the clustering results. Through feature unsupervised clustering, 78.83% (79/107) of the cases were correctly classified, which demonstrates that these features are conducive to aggressiveness classification. After sparse representation-based feature selection, 75 features are used for final testing set classification.

### predicted model

All the results of our model are shown in the Tables 2 and 3 below. It can be seen that in the cross-validation set data, the ACC of CE-T1WI, T2WI and DWI modalities alone are 0.803, 0.817 and 0.887, respectively, while the cross-validation result of the combination of the three modalities is as high as 0.930, and the sensitivity and specificity are 0.912 and 0.946, respectively. Great results were also obtained in the final independent test set. The predicted ACC of CE-T1WI, T2WI and DWI alone were 0.778, 0.778 and 0.861, respectively, while the ACC of combining the three modalities were 0.917, and the sensitivity and specificity were 0.912 and 0.946, respectively. The above results show that our proposed model combining three modalities to predict whether the thyroid is aggressive is effective.

**Table 2:** The results of cross-validation set data

Models	AUC	ACC	SEN	SPE	PPV	NPV
CE-T1WI	0.856 [0.753,0.928]	0.803 [0.711,0.891]	0.794 [0.648,0.920]	0.811 [0.621,0.913]	0.794 [0.648,0.920]	0.811 [0.618,0.914]
T2WI	0.855 [0.752,0.928]	0.817 [0.727,0.909]	0.794 [0.621,0.913]	0.838 [0.680,0.938]	0.818 [0.642,0.932]	0.816 [0.657,0.923]
DWI	0.927 [0.840,0.975]	0.887 [0.813,0.961]	0.853 [0.689,0.950]	0.919 [0.781,0.983]	0.906 [0.746,0.981]	0.872 [0.726,0.957]
Combined	0.961 [0.886,0.993]	0.930 [0.871,0.989]	0.912 [0.763,0.981]	0.946 [0.818,0.993]	0.940 [0.795,0.993]	0.921 [0.786,0.983]

95% confidence intervals are included in brackets. AUC, ACC, SEN, SPE, PPV, NPV are abbreviations of area under curve, accuracy, sensitivity, specificity, accuracy, negative predictive value and positive predictive value, respectively

**Table 3:** The results of independent test set data

Models	AUC	ACC	SEN	SPE	PPV	NPV
CE-T1WI	0.789 [0.622, 0.907]	0.778 [0.642,0.914]	0.824 [0.566,0.962]	0.737 [0.488,0.909]	0.737 [0.480,0.912]	0.824 [0.566,0.962]
T2WI	0.830 [0.668,0.934]	0.778 [0.642,0.914]	0.647 [0.383,0.858]	0.895 [0.669,0.987]	0.846 [0.546,0.981]	0.739 [0.516,0.898]
DWI	0.944 [0.813,0.993]	0.861 [0.748,0.974]	0.824 [0.566,0.962]	0.895 [0.669,0.987]	0.875 [0.605,0.986]	0.850 [0.621,0.968]
Combined	0.960 [0.836,0.997]	0.917 [0.827,1.000]	0.882 [0.636,0.985]	0.947 [0.740,0.999]	0.937 [0.686,0.999]	0.900 [0.683,0.988]

95% confidence intervals are included in brackets. AUC, ACC, SEN, SPE, PPV, NPV are abbreviations of area under curve, accuracy, sensitivity, specificity, accuracy, negative predictive value and positive predictive value, respectively

**Fig. 3:**
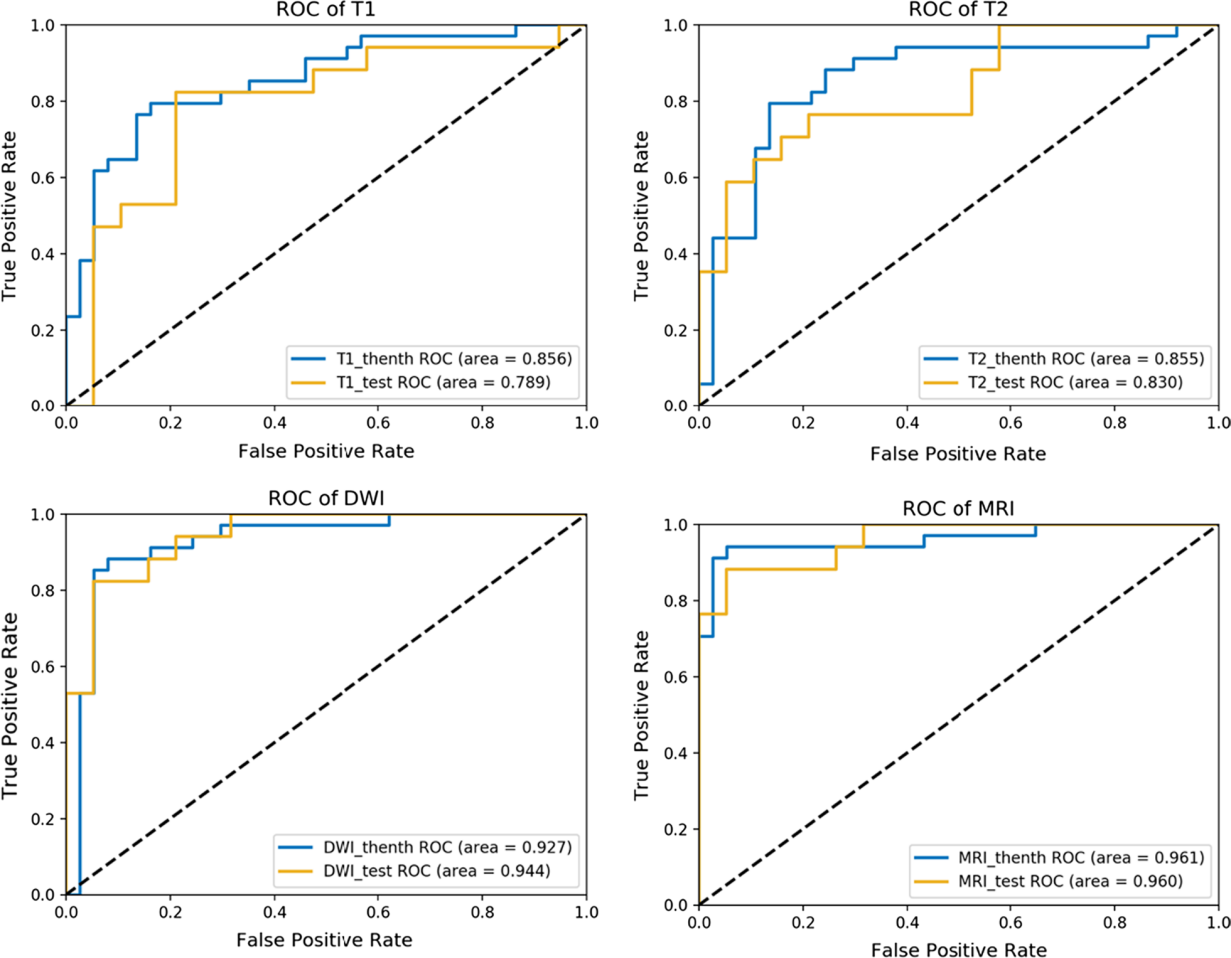
ROCs for the CE-T1WI, T2WI, DWI and combined model in predicting aggressive and non-aggressive tumors in the cross-validation and test cohort

The ROC curves based on CE-T1WI modal features, T2WI modal features, DWI modal features and the features combined three modalities at the same time are shown in Fig. 3. The blue curve represents the results of the cross validation set, and the yellow curve represents the results of the independent test set. It can also be seen from the figure that the area under the ROC curve of the combined modality is larger than that of the other three separate modalities. However, the comparisons of ROC curves based on Delong test show that the combined model is only better than T1 (*P* = 0.05) and T2 (*P* = 0.05) models alone. And the difference between the results of the combined model and the DWI model is not statistically significant (*P* = 0.70).

The combined model finally uses 75 features from three modalities to achieve an ACC of 0.917 and AUC of 0.960. Among the 75 features, there are 23 CE-T1WI modal features, 26 T2WI modal features and 26 DWI modal features, which indicates that the three modal images play an important role in the prediction of thyroid invasiveness. Among the 23 features of CE-T1WI modality, there are 2 shape features, 5 Gy features and 16 texture features. Among the 26 features of T2WI modality, there are 3 shape features, 5 Gy features and 18 texture features. Among the 26 features of DWI modality, there are 0 shape features, 10 Gy features and 16 texture features. The texture features of images play an important role in classification. These texture features describe distributions and relationships of image pixels, which can better reflect internal spatial heterogeneity of the lesions [15, 16].

**Fig. 4:**
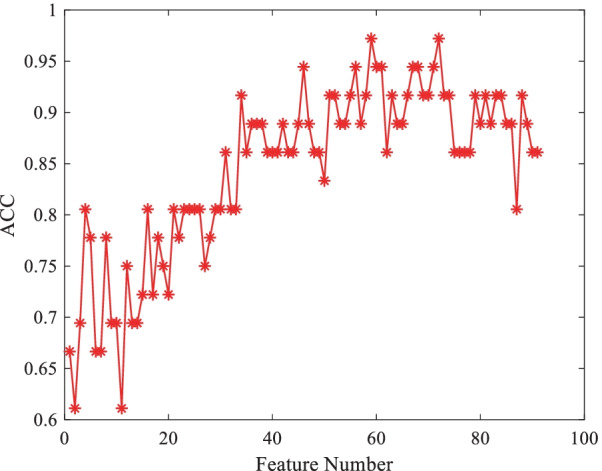
Variation of model accuracy with the number of features

**Fig. 5:**
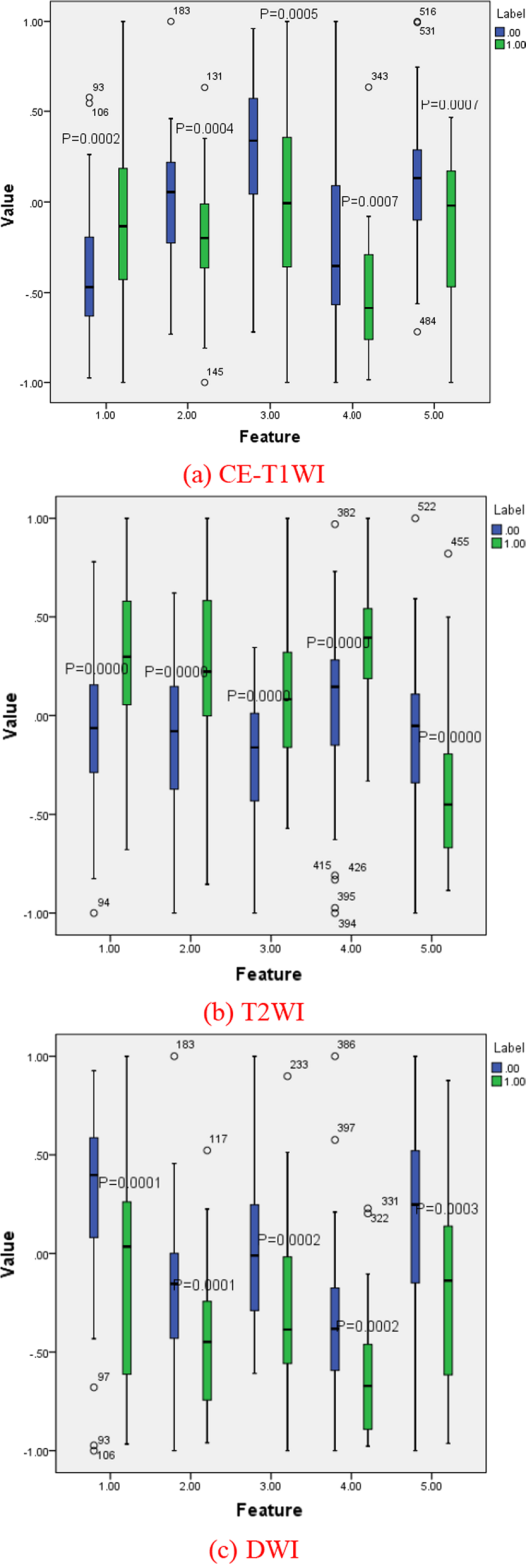
The top 5 features of importance in the classification task, CE-T1WI, T2WI and DWI, respectively

In order to further analyze the proposed model, Fig. 4 gives the change of the model classification accuracy with the increase of the number of features. It can be seen that in a certain range, the accuracy of the model increases with the increase of the number of features, which highlights the effectiveness of feature screening. With the further increase of the number of features, the accuracy of the model begins to decline, indicating that some redundant features begin to appear in the feature subset [17]. In Fig. 5 we visually compare the distribution of 5 features with the smallest P values in CE-T1WI, T2WI and DWI modalities through boxplots. The P values of t-test of these features are less than 0.001, indicating that these features have extremely significant statistical significance in classification tasks. It can also be clearly seen from the box diagram that these features of the positive and negative groups of cases are significantly different.

## Discussion

Our research shows that the machine-learning-based radiomics prediction model based on the fusion of three modalities of MRI is expected to become a noninvasive, convenient, and rapid method to evaluate the aggressiveness and non-aggressiveness of thyroid cancer. The 2015 ATA guidelines are stricter in the management of differentiated thyroid cancer, and different clinical treatment methods are adopted according to the risk assessment. Therefore, it is particularly important to comprehensively and accurately evaluate thyroid cancer before treatment. At present, the gold standard is the histopathological results of thyroid fine needle puncture, and the pathological diagnosis generally takes more than 24 h. However, using machine learning-based image analysis can predict whether it is aggressive for thyroid patients in a non-invasive and rapid way, which can not only reduce the pain of patients but also greatly shorten the diagnosis time. Therefore, the model is helpful for clinicians to design treatment methods.

MRI is widely used in tumor diagnosis because of its non-invasive and radiation-free characteristics. Based on medical image data mining technology, imaging omics quantifies tumors as high-throughput features, and then establishes the complex correlation between these features and many indicators of disease occurrence, development, and prognosis, so as to improve the accuracy of disease diagnosis and treatment efficiency. At present, the imaging research reports on thyroid cancer are mainly established on ultrasound and CT, and there are few MRI related studies. MRI has a high resolution of soft tissue density, and can accurately display the size, range, location, lymph node metastasis and the relationship with surrounding tissues and organs [18, 19]. Ma et al. [20] found that the radiological characteristics of T2WI data can predict the pathological extracapsular expansion of prostate cancer patients. DWI is the only noninvasive examination method to reflect the diffusion of living tissue at present [21]. A meta-analysis [22] shows that quantitative DWI is an accurate method to distinguish benign and malignant thyroid nodules, with noninvasive, radiation-free, sensitivity of 90% and specificity of 95%. In this paper, we investigate the performance of these three modalities and their combinations on the thyroid cancer aggressiveness prediction task.

In this study, conventional MRI sequences (CE-T1WI, T2WI) and functional imaging sequences (DWI images) were included in the study at the same time, and a multimodal imaging radiomics method was proposed to predict the aggressiveness of thyroid. Firstly, we extracted 528 high-throughput features including shape, intensity, texture and wavelet from CE-T1WI, T2WI and DWI respectively. Then, the sparse representation method was used to filter the combined 1584 features. Due to the limited number of samples in this study, the sparse representation classifier based on nonparametric training is selected for classification, so as to reduce the risk of the model overfitting [23].

Lu et al. [24] showed tumor invasiveness was evaluated by determining the ADC threshold obtained by preoperative DW-MRI (AUC, 0.85). Hu et al. [25] also used the histological characteristics of extrathyroidal extension as a tool to predict aggressiveness, showed that the AUC of the mean ADC_500_ value was 0.905, the ADC_300_ value was 0.607 and ADC_800_ values were 0.770 in differentiating ETE from without ETE (*p* < 0.001) respectively. Without the aid of ETE histological features, we directly extracted radiomics features from multimodal MRI images for modeling, and the results were better than those in [24, 25], indicating that the radiomics model based on CE-T1WI, T2WI and DWI image features has outstanding ability to predict the invasiveness of thyroid cancer. It also proved that imaging radiomics is a new non-invasive diagnostic method. Extracting high-throughput features from medical images and establishing appropriate models can be used as a tool to predict thyroid invasiveness.

There are some deficiencies in this study. Firstly, in terms of data preprocessing, ROI regions are divided manually, which is a time-consuming process. In the future, we can try to use the method of deep learning to realize automatic segmentation. Secondly, for the radiomics model, we directly splice the features extracted from each modality for the radiomics model. Although multimodal information has been applied in model prediction, it is difficult to effectively capture the deep correlation between modalities. In future work, we will establish a multimodal classifier to integrate multimodal related information in the classification process. Third, in terms of experimental data, this study only carries out experimental verification on single center data. Although we strictly divide the training and test sets, the stability and robustness of the model still need to be verified on multi center, multi parameter and multi device data sets. Therefore, in future work, we will further study the stability of multicenter data model.

## Data Availability

The datasets analyzed in this study are available from the corresponding author on request.

## Appendix 1

See Table 4.

**Table 4:** The summary of 528 features

Feature category	Feature name								Feature number
*Shape*									15
	Compactness	Compactness-square	Max-length						
	Spherical disproportion	Sphericity	Superficial-area						
	Surface to volume ratio	Volume	Region to bounding-box ratio						
	Max major-length	Min minor-length	Eccentricity						
	Orientation	Solidity	Fourier-descriptors						
*Intensity*									18
	Energy	h-energy	Kurtosis	Max					
	Mean absolute deviation	Mean	Media	Min					
	Range	Root mean square	Skewness	Standard-deviation					
	h-uniformity	variance	h-mean	h-variance					
	h-skewness	h-kurtosis							
*Texture*									39
GLCM	Energy	Contrast	Correlation	Homogeneity					
	Variance	sun average	Entropy	Dissimilarity					
	Short run emphasis		Long run emphasis						
GLRLM	Gray-level nonuniformity		Run-length nonuniformity						
	Run percentage		Low gray-level run emphasis						
	High gray-level run emphasis		Short run low gray-level emphasis						
	Short run high gray-level emphasis		Long run low gray-level emphasis						
	Long run high gray-level emphasis		Gray-level variance						
	Run-length variance		Small zone emphasis						
	Large zone emphasis								
GLSZM	Gray-level nonuniformity		Zone-size nonuniformity						
	zone percentage		Low gray-level zone emphasis						
	High gray-level zone emphasis		Small zone low gray-level emphasis						
	Small zone high gray-level emphasis		Large zone low gray-level emphasis						
	Large zone high gray-level emphasis		Gray-level variance						
	Zone-size variance								
NGTDM	Coarseness	Contrast	Busyness		Complexity		Strength		
*Wavele*									456
	LLL	HLL	LHL	HHL	LLH	HLH	LHH	HHH	
